# Impaired fasting glucose is comparable to diabetes mellitus in predicting mortality and cardiovascular events in patients undergoing peritoneal dialysis: the role of β-cell function and insulin resistance

**DOI:** 10.1080/0886022X.2026.2692758

**Published:** 2026-07-05

**Authors:** Chi-Chong Tang, Jen-Pi Tsai, Yi-Hsin Chen, Szu-Chun Hung, Yu-Li Lin, Bang-Gee Hsu

**Affiliations:** ^a^ Division of Nephrology, Hualien Tzu Chi Hospital, Buddhist Tzu Chi Medical Foundation, Hualien, Taiwan; ^b^ Institute of Medical Sciences, Tzu Chi University, Hualien, Taiwan; ^c^ School of Medicine, Tzu Chi University, Hualien, Taiwan; ^d^ Division of Nephrology, Department of Internal Medicine, Dalin Tzu Chi Hospital, Chiayi, Taiwan; ^e^ Division of Nephrology, Department of Internal Medicine, Taichung Tzu Chi Hospital, Taichung, Taiwan; ^f^ Division of Nephrology, Department of Internal Medicine, Taipei Tzu Chi Hospital, Taipei, Taiwan

**Keywords:** Peritoneal dialysis, impaired fasting glucose, diabetes mellitus, insulin resistance, β-cell function, mortality

## Abstract

Peritoneal dialysis (PD) disrupts glucose metabolism due to repeated exposure to glucose-based dialysate. This prospective cohort study evaluates whether impaired fasting glucose (IFG) confers cardiovascular and mortality risks comparable to those of diabetes mellitus (DM) and to analyze the contributions of β-cell dysfunction and insulin resistance (IR) in patients undergoing PD. 216 patients receiving PD in Taiwan were stratified by baseline glycemic status into normal fasting glucose (*n* = 71), IFG (*n* = 58), and DM (*n* = 87). β-cell function was assessed using the Homeostasis Model Assessment of β-cell Function (HOMA-β), while IR was evaluated using the Homeostasis Model Assessment of Insulin Resistance (HOMA-IR) and the triglyceride-glucose (TyG) index. The primary outcomes were all-cause mortality and 3-point major adverse cardiovascular events (3 P-MACE). Over a median follow-up of 41 months, 69 deaths (32%) occurred; 42% and 38% were attributed to cardiovascular and infectious causes, respectively. Survival curves for IFG and DM were nearly superimposable, and both were worse than that for normal glucose. In fully adjusted models, IFG independently predicted 3 P-MACE (sHR, 4.00; 95% CI, 1.50–10.66; *p* = 0.006) and all-cause mortality (HR, 2.48; 95% CI, 1.16–5.32; *p* = 0.02), with risks comparable to those observed in DM. The TyG index independently predicted both outcomes, whereas greater β-cell function was associated with a reduced risk of both endpoints. These findings suggest that cardiovascular and mortality risks in IFG are comparable to those in DM among PD patients, potentially mediated by β-cell dysfunction and increased IR.

## Introduction

Cardiovascular disease is a leading cause of mortality in patients undergoing peritoneal dialysis (PD), contributing to approximately 50% of all deaths [1,2]. The absorbed glucose from the obligate use of glucose-based dialysate solutions contributes to chronic hyperglycemia and hyperinsulinemia, triggering insulin resistance (IR) and oxidative stress that increases the cardiovascular risk in this population [3,4].

Impaired fasting glucose (IFG) is characterized by endothelial dysfunction and a progressive increase in arterial stiffness [5,6] and has emerged as an independent cardiovascular risk factor in the general population irrespective of its progression to overt diabetes mellitus (DM) [7–9]. Although the association between DM and increased mortality in patients on PD is known [10,11], it remains unclear whether the excess mortality risk associated with IFG is comparable to that of DM. This knowledge gap is particularly critical as uremia and chronic glucose absorption from dialysate use further disrupts the metabolic homeostasis in patients on PD.

IR is a key driver of atherogenesis and cardiovascular disease in the general population [12,13]. Although IR worsens as chronic kidney disease (CKD) progresses, it is further exacerbated in the PD population by continuous exposure to glucose-enriched dialysate [14,15]. IR is a robust predictor of mortality in these patients, although results vary across different IR indices. HOMA-IR is an established prognostic indicator for cardiovascular mortality among nondiabetic individuals on PD [16], while the triglyceride-glucose (TyG) index is associated with increased all-cause mortality in the PD population, regardless of DM status [17]. Additionally, CKD impairs β-cell function; this compensatory failure in a background of IR often precipitates the transition to overt DM [18–20]. However, the impact of β-cell dysfunction and IR on cardiovascular outcomes and mortality in patients on PD remain unknown.

In this prospective observational cohort study, we compared the cardiovascular morbidity and mortality risks across three glycemic categories (normal glucose, IFG, and DM) in patients on PD. Furthermore, we evaluated the longitudinal impact of glucometabolic parameters, including IR, β-cell dysfunction, serum advanced glycation end products (AGEs), PD glucose load, and icodextrin use, on these outcomes.

## Materials and methods

### Study design and population

This prospective observational study enrolled 216 adults undergoing maintenance PD at four Tzu Chi Hospitals in Taiwan (at Hualien, Dalin, Taipei, and Taichung). Patients aged ≥20 years undergoing PD vintage for at least 3 months were enrolled between February 2020 and May 2021 and were prospectively followed up until October 2024. Participants were recruited from the nephrology clinic, where the choice of dialysis modality was determined through shared decision-making and patient preference. Patients with acute infections, active malignancy, pacemakers or defibrillators, amputation of limb(s), bedridden, or those who refused to participate were excluded. Based on their glucose status as defined by the American Diabetes Association criteria [21], participants were categorized into the DM, IFG, and normal glucose groups. DM was defined by a documented medical history of DM, use of glucose-lowering medications, or fulfillment of biochemical criteria (fasting glucose ≥126 mg/dL on ≥2 occasions or HbA1c ≥6.5%). The non-DM population was further stratified into the IFG (100–125 mg/dL) and normal glucose (<100 mg/dL) groups based on their average fasting glucose levels over the previous year. The study protocol (IRB 108-219-A) was approved by the Institutional Review Board of the Buddhist Tzu Chi Medical Foundation, and written informed consent was obtained from all participants, and the study was conducted in accordance with the Declaration of Helsinki.

### Clinical and biochemical assessments

Comprehensive baseline data were extracted from electronic health records and included demographic profiles (age and sex), PD duration, and dialysis modality, including continuous ambulatory peritoneal dialysis (CAPD), automated peritoneal dialysis (APD), or continuous cycling peritoneal dialysis (CCPD). We also documented peritoneal equilibrium test results and clinical histories of hypertension and hyperlipidemia. Additionally, pharmacological data for all participants, including use of icodextrin, calcium carbonate, Calcitriol, statins, ACE inhibitors, angiotensin II receptor blockers (ARBs), calcium channel blockers and beta blockers, were obtained. Trained personnel conducted anthropometric measurements, including waist circumference, height, and weight, and obtained the blood pressure following standardized procedures. Body mass index (BMI) was determined by dividing weight in kilograms by the square of height in meters. Nutritional status was evaluated *via* the modified subjective global assessment scale, ranging from 7 (normal) to 35 (severely malnourished) [22]. The mean brachial systolic and diastolic blood pressures were recorded *via* three measurements using an automated oscillometric device.

To quantify glucose, lipid profiles (total cholesterol and triglycerides), hemoglobin, albumin, and mineral metabolism markers, including intact parathyroid hormone, calcium, and phosphorus, venous blood samples were collected after an overnight fast. Quantification of serum insulin and advanced glycation end products (AGEs) in 172 patients (80%) was conducted *via* commercial ELISA kits following the manufacturer’s instructions (Cell Biolabs, San Diego, CA, USA, and LDN, Nordhorn, Germany). HOMA-IR and HOMA-β were calculated using standard formulas as follows [23]: HOMA-IR = [fasting glucose (mg/dL) × fasting insulin (μU/mL)]/405, and HOMA-β = [360 × fasting insulin (μU/mL)]/[fasting glucose (mg/dL) − 63]. The TyG index was determined as ln [fasting triglycerides (mg/dl) × fasting glucose (mg/dl)/2] [24]. The fasting glucose result used to calculate the HOMA indices and TyG index was obtained from the same blood sample used to obtain the insulin and triglyceride levels and was not derived from the 1-year average AC-glucose. Furthermore, the daily PD glucose load was quantified based on the cumulative dextrose content in the daily dialysis prescriptions. Dialysis adequacy, which was expressed as total Kt/V, was obtained from 24-h collections of both urine and effluent dialysate, with calculations performed following standardized clinical guidelines. Renal Kt/V was used to estimate residual renal function.

### Outcome measures

The primary outcomes were 3-point major adverse cardiovascular events (3 P-MACE), which were defined as a composite of cardiovascular-related mortality, nonfatal myocardial infarction, and nonfatal stroke [25]. Clinical outcomes and specific causes of mortality were evaluated through a detailed review of electronic medical records and official death certificates and were classified as cardiovascular, infectious, cancer, trauma, or others. Patients were followed up until death, transfer to hemodialysis, kidney transplantation, or October 31, 2024, whichever occurred first.

### Statistical analysis

The normality of continuous variables was evaluated using the Kolmogorov–Smirnov test. Normally distributed variables are expressed as mean ± standard deviation, whereas skewed data are expressed as median with interquartile range. Between-group comparisons were conducted using the independent two-sample t-test for normally distributed continuous variables and the Mann–Whitney U-test for nonnormally distributed variables. Categorical data are presented as numbers (percentages), with comparisons performed *via* chi-square tests. Event-free survival probabilities were evaluated using the Kaplan–Meier method, and intergroup differences were assessed *via* the log-rank test. Cox proportional hazards regression models estimated hazard ratios for all-cause mortality, and Fine–Gray competing-risk models calculated subdistribution hazard ratios for 3 P-MACE, treating noncardiovascular deaths as competing events. Multivariable models were adjusted for age, sex, PD duration, hypertension, hyperlipidemia, BMI, waist circumference, SGA score, total Kt/V, calcium-phosphate product, hemoglobin, and albumin. HOMA-IR, HOMA-β, and AGEs, which had a nonnormal distribution, were natural log-transformed before analysis. All statistical computations were performed using IBM SPSS Statistics software, version 19.0 (IBM Corp., Armonk, NY, USA), with statistical significance defined as a two-sided P-value of 0.05.

## Results

Overall, 216 patients on PD were included in this study. The mean age was 58.4 ± 13.8 years, and 120 (55.6%) were women. Regarding glycemic status, 87 patients (40.3%) had DM, whereas 129 (59.7%) were nondiabetic. Among patients with DM, 36.8% were receiving insulin, 16.1% were receiving sulfonylureas or glinides, and 60.9% were receiving DPP-4 inhibitors. The non-DM group was further stratified into those with normal fasting glucose levels (*n* = 71; 32.9%) and those with IFG (*n* = 58; 26.9%).

Table 1 presents the baseline characteristics according to glucose status. Compared with the non-DM group, patients with DM were significantly older (61.2 ± 11.0 vs. 56.5 ± 15.2 years; *p* = 0.010) and had a shorter median PD vintage (34 vs. 56 months; *p* = 0.001). Additionally, the DM group had a higher metabolic burden as characterized by a higher weight (67.4 ± 13.9 vs 62.5 ± 14.1 kg), BMI (26.0 ± 4.0 vs. 24.2 ± 4.0 kg/m^2^; *p* = 0.001), greater waist circumference (95.2 ± 11.3 vs. 89.9 ± 10.1 cm; *p* < 0.001), and higher median systolic blood pressure (158 vs. 146 mmHg; *p* = 0.001).

**Table 1.** Clinical characteristics of patients with and without DM undergoing PD with and without DM.

Characteristics	All Patients (*n* = 216)	Non-DM (*n* = 129)	DM (*n* = 87)	*p*
**Demographics**				
Age (years)	58.4 ± 13.8	56.5 ± 15.2	61.2 ± 11.0	0.010*
Female, n (%)	120 (55.6)	76 (58.9)	44 (50.6)	0.226
PD duration (months)	48 (21–83)	56 (28–93)	34 (18–62)	0.001*
Hypertension	168 (77.8)	103 (79.8)	65 (74.7)	0.374
Hyperlipidemia	112 (51.9)	62 (48.1)	50 (57.5)	0.175
**Modality, *n* (%)**				
CAPD	82 (38)	47 (36.4)	35 (40.2)	0.573
APD or CCPD	134 (62)	82 (63.6)	52 (59.8)	
**PET status, n (%)**				
High or high average	127 (58.8)	76 (58.9)	51 (58.6)	0.966
Low or low average	89 (41.2)	53 (41.1)	36 (41.4)	
**Examination**				
Weight (kg)	64.5 ± 14.2	62.5 ± 14.1	67.4 ± 13.9	0.013*
BMI (kg/m^2^)	24.9 ± 4.1	24.2 ± 4.0	26.0 ± 4.0	0.001*
WC (cm)	92.1 ± 10.9	89.9 ± 10.1	95.2 ± 11.3	<0.001*
Modified SGA score	11 (9–12)	10 (9–12)	11 (9–13)	0.125
Systolic BP (mmHg)	151 (136–164)	146 (128–162)	158 (144–168)	0.001*
Diastolic BP (mmHg)	85 (75–93)	84 (75–96)	85 (77–91)	0.672
**Glycemic parameters**				
Fasting glucose (mg/dl)	106 (95–138)	98 (92–106)	143 (127–178)	<0.001*
Triglyceride (mg/dl)	129 (86–199)	123 (83–200)	136 (90–197)	0.286
TyG index	8.9 ± 0.8	8.7 ± 0.7	9.2 ± 0.8	<0.001*
HOMA-IR^a^	5.2 (3.3–9.1)	5.0 (3.2–8.4)	6.3 (3.4–10.3)	0.112
HOMA-β^a^	166 (96–271)	214 (134–340)	99 (62–153)	<0.001*
AGEs (ng/mL)^a^	2.5 (0.7–10.5)	2.3 (0.7–14.3)	2.5 (0.8–9.9)	0.726
PD glucose load (g/day)	136 (118–182)	136 (118–182)	136 (136–182)	0.306
**Laboratory data**				
Total cholesterol (mg/dL)	163 (134–198)	171 (142–200)	151 (126–192)	0.008*
Hemoglobin (g/dL)	9.7 (8.9–10.6)	9.6 (8.6–10.4)	10.0 (9.0–10.7)	0.016*
Albumin (g/dL)	3.6 (3.3–3.8)	3.6 (3.3–3.8)	3.6 (3.3–3.8)	0.711
Total Kt/V	2.04 (1.78–2.29)	2.07 (1.83–2.30)	1.96 (1.71–2.19)	0.027*
Renal Kt/V	0.07 (0.00–0.35)	0.01 (0.00–0.34)	0.14 (0.00–0.38)	0.055
Total Ca, corrected (mg/dL)	9.6 ± 0.8	9.6 ± 0.8	9.7 ± 0.7	0.530
Phosphorus (mg/dL)	5.0 (4.3–6.0)	5.1 (4.3–6.3)	4.9 (4.2–5.7)	0.130
Ca × P (mg^2^/dL^2^)	48 (41–58)	49 (41–61)	47 (41–56)	0.226
Intact PTH (pg/mL)	239 (91–485)	256 (109–546)	210 (80–366)	0.206
**Medications, *n* (%)**				
ACEIs or ARBs	137 (63.4)	76 (58.9)	61 (70.1)	0.094
CCBs	128 (59.3)	71 (55)	57 (65.5)	0.124
β blockers	106 (49.1)	60 (46.5)	46 (52.9)	0.359
Calcium carbonate	149 (69)	88 (68.2)	61 (70.1)	0.767
Calcitriol	48 (22.2)	33 (25.6)	15 (17.2)	0.148
Statins	67 (31)	34 (26.4))	33 (37.9)	0.071
Icodextrin	166 (76.9)	92 (71.3)	74 (85.1)	0.019*

PD, peritoneal dialysis; DM, diabetes mellitus; CAPD, continuous ambulatory peritoneal dialysis; APD, automated peritoneal dialysis; CCPD, continuous cycling peritoneal dialysis; PET, peritoneal equilibrium test; CV, cardiovascular; BMI, body mass index; WC, waist circumference; SGA, subjective global assessment; BP, blood pressure; TyG index, triglyceride-glucose index; HOMA-IR, homeostatic model assessment for insulin resistance; AGEs, advanced glycation end products; Kt/V, fractional clearance index for urea; Ca, calcium; Ca × P, calcium phosphate product; PTH, parathyroid hormone; ACEIs, angiotensin-converting enzyme inhibitors; ARBs, angiotensin receptor blockers; CCBs, calcium channel blockers; β blockers, beta blockers.

^a^HOMA-IR, HOMA-β, and AGEs were measured in 172 (80%) of the total PD population (112 non-DM and 60 DM patients).

*p < 0.05 was considered statistically significant.

Patients in the DM group had a significantly higher TyG index (median, 9.2 vs. 8.7; *p* < 0.001) and lower HOMA-β (median, 99 vs. 214; *p* < 0.001). Interestingly, HOMA-IR did not significantly differ between the DM and non-DM groups (median, 6.3 vs. 5.0; *p* = 0.112). Regarding laboratory parameters, patients with DM had lower total cholesterol levels (151 vs. 171 mg/dL; *p* = 0.008), higher median hemoglobin levels (10 vs. 9.6 g/dL; *p* = 0.016), and lower median total Kt/V (1.96 vs. 2.07; *p* = 0.027). Furthermore, icodextrin use was significantly more frequent in the DM group (85.1% vs. 71.3%; *p* = 0.019).

In the non-DM group (Table 2), those with IFG were significantly older (62.0 ± 13.2 vs. 52.1 ± 15.4 years; *p* < 0.001) and had lower median HOMA-β values (186 vs. 250; *p* = 0.006) as well as higher TyG indices (median, 8.9 vs. 8.6; *p* = 0.006). Notably, there was no significant difference in HOMA-IR between the IFG and normal glucose groups (median, 5.0 vs. 5.2; *p* = 0.505). The daily peritoneal glucose load was significantly lower in the IFG group than in the normal glucose group (median, 136 vs. 145, *p* = 0.039).

**Table 2.** Comparisons of patients on PD with normal and impaired fasting glucose.

Characteristics	Fasting glucose		*p*
	Normal (*n* = 71)	Impaired (*n* = 58)	
**Demographics**			
Age (years)	52.1 ± 15.4	62.0 ± 13.2	<0.001*
Female, n (%)	41 (57.7)	35 (60.3)	0.765
PD duration (months)	53 (28–83)	57 (26–105)	0.436
Hypertension	59 (83.1)	44 (75.9)	0.308
Hyperlipidemia	35 (49.3)	27 (46.6)	0.756
**Modality, *n* (%)**			
CAPD	23 (32.4)	24 (41.4)	0.291
APD or CCPD	48 (67.6)	34 (58.6)	
**PET status, n (%)**			
High or high average	39 (54.9)	37 (63.8)	0.309
Low or low average	32 (45.1)	21 (36.2)	
**Examination**			
Weight (kg)	63.1 ± 14.3	61.7 ± 13.8	0.581
BMI (kg/m^2^)	24.2 ± 4.1	24.2 ± 3.9	0.942
WC (cm)	89.5 ± 10.3	90.5 ± 9.9	0.606
Modified SGA score	10 (9–12)	11 (9–13)	0.225
Systolic BP (mmHg)	148 (127–163)	146 (130–162)	0.813
Diastolic BP (mmHg)	84 (75–96)	87 (73–96)	0.538
**Glycemic parameters**			
Fasting glucose (mg/dL)	92 (90–96)	107 (103–116)	<0.001*
Triglyceride (mg/dL)	111 (78–187)	138 (88–214)	0.104
TyG index	8.6 ± 0.6	8.9 ± 0.7	0.006*
HOMA-IR ^a^	5.2 (2.6–8.4)	5.0 (3.7–8.3)	0.505
HOMA-β^a^	250 (166–401)	186 (104–260)	0.006*
AGEs (ng/mL)^a^	3.3 (0.7–27.5)	1.8 (0.6–7.5)	0.138
PD glucose load (g/day)	145 (125–182)	136 (102–165)	0.039*
**Laboratory data**			
Total cholesterol (mg/dL)	148 (146–200)	172 (135–199)	0.910
Hemoglobin (g/dL)	9.8 (8.6–10.7)	9.3 (8.5–10.0)	0.081
Albumin (g/dL)	3.6 (3.3–3.8)	3.6 (3.3–3.9)	0.547
Total Kt/V	2.05 (1.83–2.29)	2.11 (1.79–2.33)	0.818
Renal Kt/V	0.03 (0.00–0.35)	0.00 (0.00–0.28)	0.338
Total Ca, corrected (mg/dL)	9.6 ± 0.8	9.6 ± 0.7	0.943
Phosphorus (mg/dL)	5.2 (4.6–6.8)	4.9 (4.3–5.9)	0.044*
Ca × P (mg^2^/dL^2^)	49.8 (42.5–67.3)	47.6(38.6–56.9)	0.081
Intact PTH (pg/mL)	291 (118–611)	254 (82–457)	0.291
**Medications, *n* (%)**			
ACEIs or ARBs	44 (62)	32 (55.2)	0.435
CCBs	37 (52.1)	34 (58.6)	0.460
β blockers	33 (46.5)	27 (46.6)	0.993
Calcium carbonate	50 (70.4)	38 (65.5)	0.552
Calcitriol	19 (26.8)	14 (24.1)	0.734
Statins	16 (22.5)	18 (31.0)	0.276
Icodextrin	46 (64.8)	46 (79.3)	0.070

PD, peritoneal dialysis; DM, diabetes mellitus; CAPD, continuous ambulatory peritoneal dialysis; APD, automated peritoneal dialysis; CCPD, continuous cycling peritoneal dialysis; PET, peritoneal equilibrium test; CV, cardiovascular; BMI, body mass index; WC, waist circumference; SGA, subjective global assessment; BP, blood pressure; TyG index, triglyceride-glucose index; HOMA-IR, homeostatic model assessment for insulin resistance; HOMA-β, homeostasis model assessment of β-cell function; AGEs, advanced glycation end products; Kt/V, fractional clearance index for urea; Ca, calcium; Ca × P, calcium phosphate product; PTH, parathyroid hormone; ACEIs, angiotensin-converting enzyme inhibitors; ARBs, angiotensin receptor blockers; CCBs, calcium channel blockers; β blockers, beta blockers.

^a^HOMA-IR, HOMA-β, and AGEs were measured in 112 (85%) non-DM patients.

*p < 0.05 was considered statistically significant.

**Figure 1.**
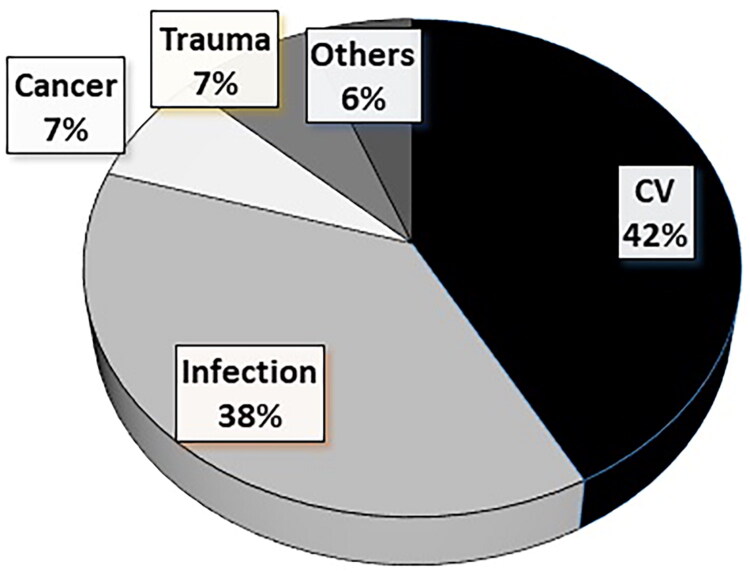
Causes of mortality among the 69 deceased patients during a median follow-up period of 41 months.

**Figure 2.**
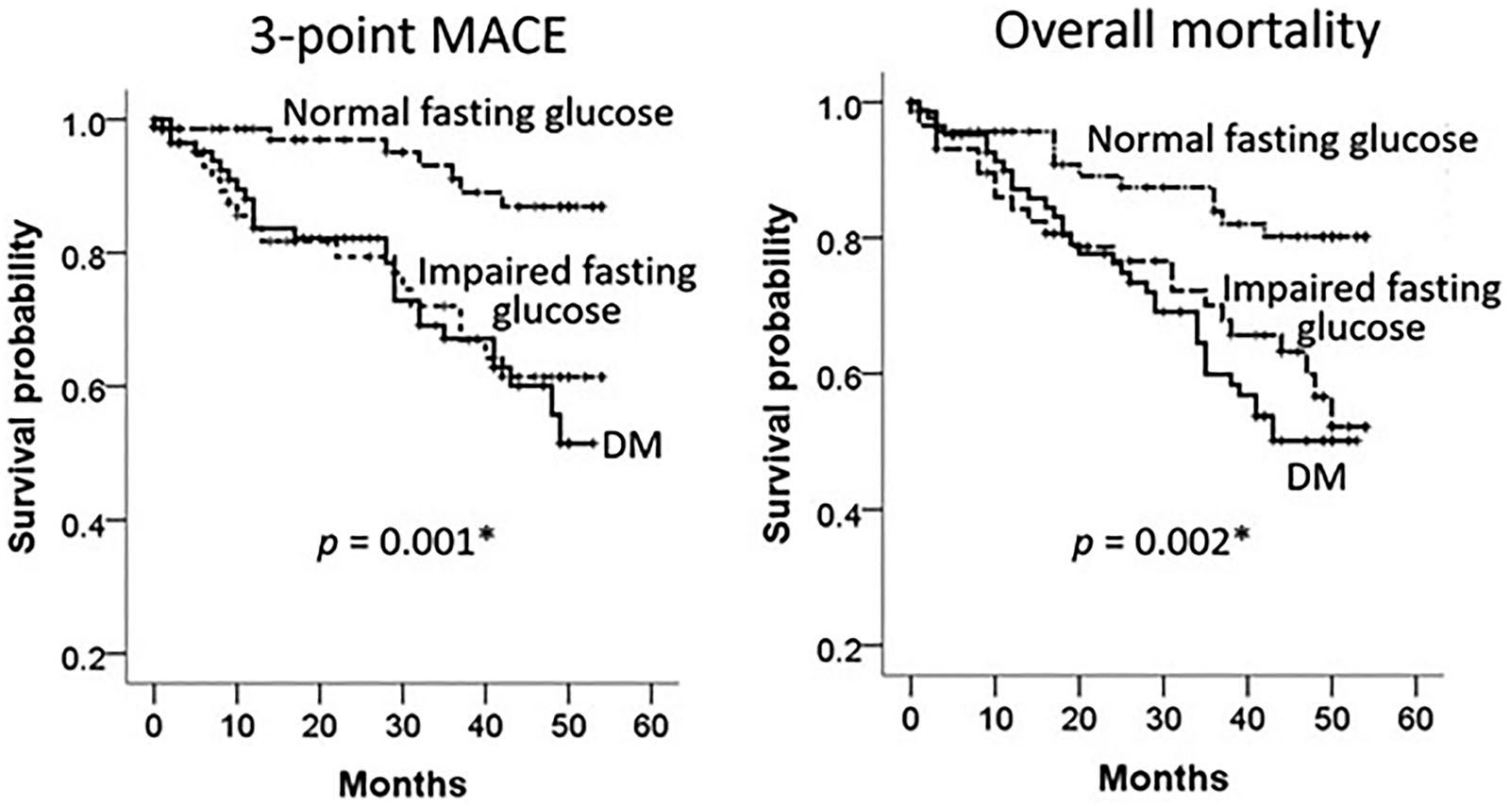
Kaplan–Meier survival curves for 3-point major adverse cardiovascular events (MACE) and all-cause mortality according to glycemic status (normal glucose, impaired fasting glucose, and diabetes mellitus).

There were 69 deaths (32%) during a median follow-up of 41 months; 29 (42%) due to cardiovascular causes, 26 (38%) due to infection, and 14 (20%) due to other causes (Figure 1). Compared with the normal glucose group, Kaplan–Meier analysis revealed significantly reduced 3 P-MACE-free survival and overall survival in both the IFG and DM groups (log-rank *p* = 0.001 and *p* = 0.002, respectively; Figure 2). Notably, the survival curves for the IFG and DM groups were nearly superimposable.

The association of normal glucose, IFG, and DM on the risk of 3-point MACE and overall mortality is presented in Table 3. After adjustment, multivariable analysis revealed that IFG independently predicted 3 P-MACE (sHR 4.00; 95% CI, 1.50–10.66; *p* = 0.006) and all-cause mortality (HR 2.48; 95% CI, 1.16–5.32; *p* = 0.02) compared to normal fasting glucose. DM showed comparable risks, with a sHR of 2.66 (95% CI, 1.13–6.23; *p* = 0.03) for 3 P-MACE and HR of 2.25 (95% CI, 1.13–4.48; *p* = 0.02) for all-cause mortality.

**Table 3.** Association of normal glucose, impaired fasting glucose, and DM on the risk of 3-point MACE and overall mortality among patients undergoing PD.

Variables	3-point MACE			
	Unadjusted		Adjusted	
	SHR (95% CI)^¶^	*p*	SHR (95% CI)^¶^	*p*
Normal fasting glucose levels	Reference		Reference	
Impaired fasting glucose	3.66 (1.54, 8.68)	0.003*	4.00 (1.50, 10.66)	0.006*
Diabetes Mellitus	3.73 (1.64, 8.49)	0.002*	2.66 (1.13, 6.23)	0.025*

**Table:** 

Variables	Overall mortality			
	HR (95% CI)	*p*	HR (95% CI)	*p*
Normal fasting glucose levels	Reference		Reference	
Impaired fasting glucose	2.55 (1.26, 5.15)	0.009*	2.48 (1.16, 5.32)	0.020*
Diabetes Mellitus	3.00 (1.56, 5.79)	0.001*	2.25 (1.13, 4.48)	0.021*

In the adjusted model, age, sex, PD duration, hypertension, hyperlipidemia, BMI, waist circumference, modified SGA score, total Kt/V, Ca × P product, Hb, and Albumin were adopted as covariates.

**^¶^**Fine and Gray models were used to estimate subdistribution hazard ratios (SHRs) accounting for competing risk of non-CV death.

*p < 0.05 was considered statistically significant.

Further exploration of the associations between glucometabolic parameters, 3-point MACE, and all-cause mortality is presented in Table 4. In fully adjusted models, the TyG index independently predicted 3 P-MACE (sHR 1.66; 95% CI, 1.07–2.58; *p* = 0.02) and all-cause mortality (HR 1.59; 95% CI, 1.13–2.24; *p* = 0.009). Meanwhile, HOMA-IR was not significantly associated with either outcome. In contrast, higher log-HOMA-β was associated with reduced risks of 3 P-MACE (sHR, 0.37; 95% CI, 0.14–0.94; *p* = 0.04) and all-cause mortality (HR, 0.28; 95% CI, 0.12–0.66; *p* = 0.004). AGEs, peritoneal glucose load, and icodextrin use did not predict these outcomes.

**Table 4.** Association of glucometabolic parameters with 3-point MACE and all-cause mortality in patients on PD.

Variables	3-point MACE			
	Unadjusted		Adjusted	
	SHR (95% CI)^¶^	*p*	SHR (95% CI)^¶^	*p*
TyG index	1.45 (1.04, 2.02)	0.027*	1.66 (1.07, 2.58)	0.023*
Log-HOMA-IR^a^	1.02 (0.35, 2.99)	0.970	0.83 (0.27, 2.53)	0.740
Log-HOMA-β^a^	0.25 (0.12, 0.52)	<0.001*	0.37 (0.14, 0.94)	0.036*
Log-AGEs (ng/mL)^a^	1.15 (0.89, 1.48)	0.280	1.17 (0.86, 1.60)	0.320
PD glucose load (g/day)	1.00 (0.99, 1.00)	0.440	1.00 (0.99, 1.00)	0.220
Icodextrin	1.48 (0.73, 3.00)	0.280	1.53 (0.64, 3.64)	0.340

**Table:** 

Variables	Overall mortality			
	HR (95% CI)	*p*	HR (95% CI)	*p*
TyG index	1.43 (1.06, 1.94)	0.020*	1.59 (1.13, 2.24)	0.009*
Log-HOMA-IR^a^	1.21 (0.49, 2.96)	0.683	0.83 (0.31, 2.24)	0.709
Log-HOMA-β^a^	0.26 (0.12,0.56)	0.001*	0.28 (0.12, 0.66)	0.004*
Log-AGEs (ng/mL)^a^	0.89 (0.70, 1.12)	0.306	0.87 (0.66, 1.15)	0.321
PD glucose load (g/day)	1.00 (0.99, 1.00)	0.228	1.00 (0.99, 1.00)	0.136
Icodextrin	1.16 (0.65, 2.09)	0.613	0.91 (0.48, 1.73)	0.768

In the adjusted model, age, sex, PD duration, DM, hypertension, hyperlipidemia, BMI, waist circumference, modified SGA score, total Kt/V, Ca × P product, Hb, and Albumin were adopted as covariates.

**^¶^**Fine and Gray models were used to estimate subdistribution hazard ratios (SHRs) accounting for competing risk of non-CV death.

^a^ HOMA-IR, HOMA-β, and AGEs were measured in 172 (80%) of the total PD population.

*p < 0.05 was considered statistically significant.

## Discussion

In this prospective cohort study, we revealed that IFG in patients with PD confers cardiovascular and mortality risks comparable to having DM. The TyG index was a prognostic indicator of adverse outcomes, whereas preserved β-cell function, as reflected by a higher HOMA-β, was associated with a lower risk of adverse outcomes. These findings underscore the need to pay special attention to patients on PD with IFG and to incorporate evaluations on insulin resistance and β-cell dysfunction in this population.

The association between preexisting and incident DM and mortality in patients with PD is well established [10,11,26,27]. However, in clinical practice, the importance of IFG is often underrecognized. A prior study of 362 patients with PD reported that nondiabetic patients with IFG had a higher 2-year all-cause mortality (HR 2.719), similar to our results (HR 2.48) [28]. Kaplan–Meier survival analysis also showed that mortality outcomes in the IFG group were comparable to those in the DM group [28]. In our study, we further extended these observations by demonstrating that the risks of 3 P-MACE were higher in the IFG group than in the normal glucose group and comparable to those in the DM group. This increased risk associated with IFG reflects PD-specific factors. Chronic exposure to glucose-containing dialysate increases systemic glucose absorption, promoting IR and systemic inflammation and creating an adverse metabolic environment that causes vascular damage [3,29–31]. Regardless of DM status, a fasting blood glucose >100 mg/dL was reportedly associated with a higher mortality risk [32]. Our earlier work also demonstrated that in nondiabetic patients with PD, IFG, IR, and PD glucose load were closely associated with aortic stiffness [33]. This study further demonstrated the impact of IR and β-cell function on cardiovascular outcomes and mortality risk.

As a surrogate marker of IR, the TyG index predicts mortality in patients with PD; each 1-unit increase is associated with all-cause mortality (HR 1.62) and is align with our results (HR 1.59) [34]. Compared with other IR indices such as TyG-BMI and the TG/HDL-C ratio, the TyG index also demonstrated the greatest predictive stability for all-cause mortality [17]. Our study also evaluated the HOMA-IR for comparison, revealing that it was not independently associated with the primary outcomes. The advantage of the TyG index as a surrogate of IR in patients on PD is explained by its superior ability in reflecting glucose-lipid dysmetabolism, including impaired triglyceride clearance and increased hepatic synthesis, which are often exacerbated by glucose absorption from dialysate solutions [35–37]. Furthermore, the TyG index is reportedly superior to HOMA-IR in predicting metabolic syndrome [38,39]. As metabolic syndrome is highly prevalent in PD populations, this may account for its superior prognostic performance. Another study reported that HOMA-IR can predict cardiovascular morbidity and mortality; however, it included only nondiabetic patients on PD, which may explain why its findings are inconsistent with ours [16]. This emphasis on IR rather than diabetes status is also observed in other dialysis modalities. A previous study demonstrated that among hemodialysis patients, glucose tolerance does not differ substantially between diabetic and nondiabetic individuals but is instead primarily driven by IR [40]. This finding highlights the critical role of IR in glucose disposal across the entire dialysis population.

The protective association of higher HOMA-β in patients on PD deserves emphasis. In nondiabetic cohorts, β-cell dysfunction is associated with cardiovascular events [41,42]. Our study extends these observations to patients on PD, demonstrating that higher HOMA-β is associated with a lower risk of both MACEs and all-cause mortality. Although the mechanisms of the impact of β-cell dysfunction to cardiovascular risk in patients on PD are unclear, several pathways have been proposed. CKD can impair β-cell function by increasing urea levels, which reduce glucose utilization and insulin secretion [18]. Furthermore, β-cell dysfunction is associated with autonomic dysfunction and reduced heart rate variability in individuals with coronary artery disease irrespective of IR [43,44]. Collectively, these findings suggest that β-cell dysfunction may further increase the risk of cardiovascular complications and mortality among patients on PD.

This study has several strengths. Data directly comparing the clinical outcomes between patients with IFG and DM within the PD population are limited, and this study revealed that both groups face comparable cardiovascular outcomes and mortality risks. Additionally, our findings establish the TyG index as a prognostic indicator superior to HOMA-IR while simultaneously underscoring the protective clinical value of preserved β-cell function as evaluated by HOMA-β. Finally, the use of a larger and more inclusive cohort from four hospitals across Taiwan strengthens the statistical power and clinical generalizability of our results compared to previous small-sample research. However, this study also has several limitations. First, as an observational study, causal inferences could not be established. Second, the HOMA equation was used to assess β-cell function and IR, but it is based on steady metabolic state assumptions and may not fully reflect how insulin is dynamically secreted. Third, HOMA-IR, HOMA-β, and AGEs were obtained in only 172 patients (approximately 80%), potentially introducing selection bias. Fourth, survival bias cannot be excluded as patients with DM with severe complications or significant functional impairment may have been less likely to be enrolled, potentially leading to a relatively healthier DM population and an underestimation of mortality risk in the DM group. Finally, the potential influence of unmeasured confounders, including erythropoietin dosage, dietary patterns, physical activity levels, and markers of systemic inflammation, could not be excluded.

In conclusion, IFG in patients on PD confers cardiovascular and mortality risks comparable to those as having DM. As assessed by the TyG index, IR predicts poor outcomes, whereas higher HOMA-β is associated with lower risks of MACE and overall mortality. Early detection of IFG, along with routine evaluations of the TyG index and β-cell function, may facilitate risk stratification. From a clinical perspective, strategies aimed at reducing daily glucose exposure from dialysate solutions and increasing the use of icodextrin may be considered, particularly in patients with IFG. Future studies should explore whether interventions targeting IR and β-cell preservation could improve outcomes in this population.

## Data Availability

The data presented in this study are available on request from the corresponding author.
